# Evidence-Based Strategies for the Treatment of Peritoneal Malignancies during Health Care Resource Restriction: The COVID-19 Pandemic

**DOI:** 10.3390/curroncol28010006

**Published:** 2020-12-01

**Authors:** Farhana Shariff, Danielle Bischof, Anand Govindarajan, Rebecca Prince, Ronald Burkes, Erika Haase, Lloyd Mack, Walley Temple, Pamela Hebbard, Cindy Boulanger-Gobeil, Carman Giacomantonio, Alexandre Brind’Amour, Lucas Sidéris, Pierre Dubé, Trevor Hamilton, Andrea MacNeill, Antoine Bouchard-Fortier, Rami Younan, Andrea McCart

**Affiliations:** 1 University Health Network, Toronto, ON M5G 1L7, Canada; Danielle.Bischof@sinaihealth.ca (D.B.); Anand.Govindarajan@sinaihealth.ca (A.G.); Rebecca.Prince@uhn.ca (R.P.); ron.burkes@sinaihealth.ca (R.B.); 2 Division of Surgical Oncology, Department of Surgery, University of Toronto, Toronto, ON M5G 1L7, Canada; 3 Mount Sinai Hospital, Toronto, ON M5G 1L7, Canada; 4 Division of Medical Oncology, Department of Medicine, University of Toronto, Toronto, ON M5G 1L7, Canada; 5 Department of Surgery, University of Alberta, Edmonton, AB T6G 2R3, Canada; ehaase@ualberta.ca; 6 Department of Surgery and Oncology, University of Calgary, Calgary, AB T2N 1N4, Canada; lloyd.mack@albertahealthservices.ca (L.M.); walley.temple@albertahealthservices.ca (W.T.); abfortier@gmail.com (A.B.-F.); 7 Department of Surgery, University of Manitoba, Cancer Care Manitoba, Winnipeg, MN R3T 2N2, Canada; pamelahebbard@gmail.com; 8 CHU de Québec, Département de Chirurgie, Université Laval, Quebec City, QC G1V 4G2, Canada; cindy.boulanger-gobeil@chudequebec.ca; 9 Department of Surgery, Dalhousie University, Halifax, NS B3H 4R2, Canada; carman.giacomantonio@nshealth.ca; 10 Maisonneuve-Rosemont Hospital, Montréal, QC H1T 2M4, Canada; alexandre.brindamour.1@ulaval.ca (A.B.); lucas.sideris@umontreal.ca (L.S.); pdube1234@gmail.com (P.D.); 11 Department of Surgery, University of British Columbia, Vancouver, BC V5Z 1M9, Canada; Trevor.Hamilton@vch.ca (T.H.); Andrea.MacNeill@vch.ca (A.M.); 12 Centre Hospitalier de l’Université de Montréal, Montréal, QC H2X 3E4, Canada; rami.younan.chum@ssss.gouv.qc.ca

**Keywords:** peritoneal malignancies, pandemic triage strategies

## Abstract

*Background:* The COVID-19 pandemic has put enormous pressure on hospital resources, and has affected all aspects of patient care. As operative volumes decrease, cancer surgeries must be triaged and prioritized with careful thought and attention to ensure maximal benefit for the maximum number of patients. Peritoneal malignancies present a unique challenge, as surgical management can be resource intensive, but patients have limited non-surgical treatment options. This review summarizes current data on outcomes and resource utilization to help inform decision-making and case prioritization in times of constrained health care resources. *Methods:* A rapid literature review was performed, examining surgical and non-surgical outcomes data for peritoneal malignancies. Narrative data synthesis was cross-referenced with relevant societal guidelines. Peritoneal malignancy surgeons and medical oncologists reviewed recommendations to establish a national perspective on case triage and mitigating treatment strategies. *Results and Conclusions:* Triage of peritoneal malignancies during this time of restricted health care resource is nuanced and requires multidisciplinary discussion with consideration of individual patient factors. Prioritization should be given to patients where delay may compromise resectability of disease, and where alternative treatment options are lacking. Mitigating strategies such as systemic chemotherapy and/or surgical deferral may be utilized with close surveillance for disease stability or progression, which may affect surgical urgency. Unique hospital capacity, and ability to manage the complex post-operative course for these patients must also be considered to ensure patient and system needs are aligned.

## 1. Introduction

The COVID-19 pandemic as declared by the World Health Organization, is a health crisis that has placed extraordinary pressures on health care systems around the globe [1]. As a result, factors including high disease prevalence, limited hospital resources, risk of nosocomial spread, and risk of disease transmission to health care workers in the operating room (OR) have contributed to postponement of a large number of elective operative cases [1,2,3]. This decline in operative volume has necessitated new systems for triaging and prioritizing surgical cases, most notably for cancer patients [4]. As the pandemic evolves, subspecialty groups are working to provide resources to support decision-making and case triage for various pathologies [5,6,7,8,9], aiming to provide maximal patient care, while mitigating risks to patients, health-care workers, and already strained systems. While many surgeries will undoubtedly be delayed during the peak of health system stress, delays in cancer surgery have the potential to affect overall prognosis and lifespan. Accordingly, patient triage and prioritization decisions require the utmost thoughtfulness and attention [7]. 

Peritoneal malignancies include tumours of both primary peritoneal and non-peritoneal visceral etiologies [10]. Treatment algorithms for these complex disseminated diseases vary between underlying pathologies, but generally include a combination of locoregional treatments such as cytoreductive surgery (CRS)—with or without concurrent hyperthermic intraperitoneal chemotherapy (HIPEC)—and systemic chemotherapeutic options when available. Unique considerations for peritoneal malignancies in the context of the current pandemic include the potentially high resource utilization by these patients, balanced with the availability, or lack, of alternative treatment strategies. 

Although interactions between COVID-19 and surgical risk are still not well understood, early observations from China demonstrate poorer outcomes in afflicted surgical patients. In a retrospective analysis of 34 patients who were asymptomatic at presentation but diagnosed with COVID-pneumonia post-operatively, 44% of patients required ICU admission—almost double the rate of those not undergoing surgical intervention—and 20% died [11]. As an added complexity, early data suggests COVID-specific morbidity in cancer patients, including critical care admission, mechanical intubation, and even mortality is up to 3.5× higher than those without underlying cancer diagnoses [4,12]. While the number of patients in these reports is small, these early results speak to the increased risks cancer patients face from COVID-19 infection, which must be balanced against the risk of under-treatment or delays in treatment of their underlying pathologies. Potentially complicating the issue further, it is unknown whether aerosolizing procedures such as laparoscopy, or peritoneal perfusion may increase COVID transmission risk to health care practitioners [13].

The Society of Surgical Oncology, Cancer Care Ontario, and the National Comprehensive Cancer Network are just a few professional organizations that have provided expert consensus documents to help practitioners triage cases when necessary [5,6,9,14]. The majority of these recommendations, however, provide broad guidance regarding case prioritization, with minimal emphasis on patient and disease specific decision-making. As relatively rare conditions, triage for peritoneal malignancies are not extensively discussed in these guidance documents [5,14]. The complex process of case triage, which hospitals are grappling with, requires physicians to have an understanding of the evidence regarding treatment benefits, potential complications that may burden an already strained health care system, individual patient factors and availability of alternative treatment strategies. Such knowledge is essential for case prioritization, distribution of resources, and to enable evidence-informed conversations with patients. 

This review aims to summarize data regarding resource utilization, as well as disease recurrence and progression for complex peritoneal diseases of non-gynecologic origin to help guide resource allocation and decision-making during a time of strained and restricted operating room (OR) and critical care resources.

## 2. Methods

### 2.1. Literature Sources

A targeted rapid literature review [15] using PUBMED and MEDLINE was performed, identifying publications reporting on outcomes for peritoneal malignancies with surgical and/or non-surgical interventions. Evidence discussing short-term morbidity, resource utilization, and predictors of oncologic outcome was examined. Citation tracking and manual reference list examination were performed to screen for additional studies. Recommendations and guidelines from oncologic societies in North America as of April 11/2020 were consulted to identify and compare current expert consensus with data extracted for this review. 

### 2.2. Inclusion and Exclusion Criteria

English language papers between years 2000–2020, which were available in full article form, and discussed treatment outcomes for peritoneal malignancies were included. Feasibility-only studies of treatment strategies were excluded. Due to heterogeneity in levels of evidence and the relatively small volume of literature, all relevant randomized studies, cohort studies, case series, and retrospective data reviews were included. 

### 2.3. Data Synthesis

F.S. performed literature search and data abstraction, and D.B./A.M./A.G., peritoneal surgeons at Mt Sinai Hospital agreed upon data inclusion and recommendations. Due to heterogeneity in study quality and treatment strategy, a narrative summary approach was selected, and data was not pooled for further analysis. Data were examined and summarized by disease process, and divided by treatment strategy (i.e., observation, surgical management, medical management, combination management) when relevant.

A review of data and recommendations was performed by two medical oncologists from the peritoneal disease program at Mount Sinai Hospital to ensure appropriate multidisciplinary perspectives. Finally, data synthesis and recommendations were reviewed and accepted by peritoneal surgeons from 8 complex peritoneal malignancy programs in Canada, achieving agreement and consensus on a national level. A summary of major conclusions can be found in Table 1.

## 3. Triage and Treatment Strategies

### Appendiceal Mucoceles

The term *appendiceal mucocele* refers to dilatation of the appendix with mucinous content, which may be the result of a variety of causes including chronic appendicitis/inflammatory mucoceles, appendiceal diverticula, and benign or malignant appendiceal neoplasms. While size alone is not predictive of underlying etiology, mucoceles less than 2 cm in diameter are less likely to be associated with neoplastic causes [16,17]. These have been included in this review, as rupture portends the potential for peritoneal spread when appendiceal neoplasms are the underlying etiology.

At present, there is no clear evidence regarding likelihood of mucocele rupture over a particular time course. Within neoplastic causes, low-grade appendiceal mucinous neoplasms (LAMNs) are generally chronic and indolent in their progression [18]. In contrast, invasive lesions such as adenocarcinoma have potential to progress more quickly both within the appendix itself as well as locoregionally and to distant organs [19]. The decision to pursue operative intervention for mucoceles in the time of pandemic conditions should be guided by the specific stresses of an individual hospital or health care region. If Operating Room (OR) access is limited, deferral is reasonable to consider given their relatively slow growing nature. In contrast, if hospital beds and Intensive Care Unit (ICU) resources are the primary concern, resection of the mucocele may be reasonable, as these can often be resected laparoscopically, with same day discharge. Where these cases are postponed, close surveillance with cross-sectional imaging should be performed to ensure mucocele stability, as features such as progression in size, or suspicious nodal findings may suggest more aggressive pathology and need for expedited resection. 

## 4. Pseudomyxoma Peritonei (PMP)

The term pseudomyxoma peritonei describes a clinical syndrome consisting of mucinous ascites secondary to perforation and peritoneal dissemination of mucinous neoplasms primarily of the appendix (most commonly low-grade mucinous neoplasms—LAMNs) [19]. The current standard treatment for PMP originating from a LAMN is cytoreduction of peritoneal disease followed by intraperitoneal perfusion of hyperthermic chemotherapy. CRS/HIPEC is associated with significant potential peri-operative morbidity and increased length of stay [20,21], which must be considered when surgical and intensive post-operative resources may be limited.

### 4.1. Observation

Retrospective data from Zih et al. (2014), examined overall (OS) and progression free survival (PFS) in PMP patients with limited, low-grade disease, and minimal symptoms, managed expectantly with clinical and imaging surveillance [22]. Five-year PFS in this population was 82%; in those who progressed, median time to disease progression was 50 months, with no compromise in the ability to achieve adequate cytoreduction [22]. These results speak to the indolent nature of this pathology, and support the safety of surgical delay with close observation in patients meeting the above criteria. 

A theoretical risk of observation in patients with PMP from low-grade appendiceal neoplastic disease is one of malignant dedifferentiation and progression to more invasive disease. Tumour analysis after repeat CRS for recurrent PMP by Chua et al [23] found 9 of 58 (15%) patients underwent malignant dedifferentiation of the primary tumour—four into well differentiated mucinous adenocarcinoma, and five into moderately differentiated mucinous adenocarcinoma. While 5-year survival rates in these patients were lower (75% vs. 89% from time of diagnosis), median time from diagnosis of PMP to disease progression was 41 months [23]. Despite the risk of tumour dedifferentiation, this speaks to the relative safety of expectant management if required in the context of a time-limited health resource crisis, as the likelihood of progression to “unsalvageable” disease within a short timeframe of 3–6 months is exceedingly low.

### 4.2. Cytoreductive Surgery (CRS) and Hyperthermic Intraperitoneal Chemotherapy (HIPEC)

CRS alone as a management approach results in a median OS of up to 10 years [24]. Median progression free survival from CRS surgery alone, however, is between 24–30 months [25], with a median time to recurrence of 24 months, even in patients with complete or near-complete cytoreduction (CC0—no visible peritoneal disease after cytoreduction, or CC1—nodules < 2.5 mm after cytoreduction) [26]. 

Over the past decade, an increasing number of large case series examining outcomes with the addition of HIPEC have been published. Although intraperitoneal chemotherapy regimens differed, 5-year disease free survival from PMP following CRS/HIPEC of 31–74% have been reported, with better results and median progression free survival of 8 years in patients who had complete (CC0) or near-complete cytoreduction (CC1) [25,27,28,29].

At present, no randomized trials comparing CRS alone versus CRS/HIPEC for PMP exist [22]. Of note, multivariate analyses performed in a large retrospective study by Chua et al. (2012) demonstrated association between HIPEC and improved progression-free survival [23]. Acknowledging the indolent nature of this disease, and a need in many cases for extensive cytoreduction, PMP cases should be triaged based on site-specific challenges. In cases where maintaining higher-level ICU and monitored care capacity is of concern, definitive CRS and HIPEC for asymptomatic or minimally symptomatic patients can be delayed. As with mucoceles, these patients should undergo close surveillance for more rapid progression, which may suggest mixed or dedifferentiated tumour type requiring more urgent intervention. Given the low rates of response to systemic chemotherapy in PMP patients, and lack of alternative non-surgical treatment options [30]; those with progressive disease on surveillance, borderline resectability and large Krukenberg lesions causing symptoms should be prioritized for surgery. Additionally, patients with significant symptomatology related to intraabdominal disease burden, such as diaphragmatic compression, partial bowel obstruction, and renal failure should be considered for temporizing debulking surgery, which may improve symptoms with minimal operative morbidity [31], and serve as a bridge to definitive surgical therapy if eligible.

## 5. Colorectal Cancer (CRC) with Peritoneal Metastases

Peritoneal metastatic disease from colon and rectal cancer (M1c disease), with or without visceral organ metastases is estimated to occur in 8–10% of colorectal cancer patients [32]. Patients with peritoneal metastasis of colorectal origin differ from PMP patients due to the more aggressive and progressive nature of disease. In contrast to pseudomyxoma, delayed treatment of these patients may carry a risk for disease progression, and subsequent surgical unresectability [32]. As a consequence, this group of patients generally requires more urgent prioritization over more indolent peritoneal pathologies such as PMP.

### 5.1. Systemic Chemotherapy

A recent systematic review by Waite and Youssef (2017) [33] suggested no clear evidence that neoadjuvant chemotherapy improves overall survival in patients with peritoneal metastases from CRC going on to surgical intervention. This study was limited, however, by significant heterogeneity between studies, all of which were non-randomized and/or retrospective. At present, the current standard treatment in Canada consists of neoadjuvant 5-fluorouracil-based chemotherapy for most cases of colorectal cancer with peritoneal metastases [34], with the potential benefits of tumour downstaging, improvement in the completeness of cytoreduction, and treatment of micrometastases. Most importantly, response to chemotherapy may provide a useful marker of disease biology, which may help guide decisions about whether aggressive surgical intervention should be pursued [33]. In times of pandemic, patients demonstrating disease stability or improvement on systemic chemotherapy should continue treatment as tolerated until operating room and critical care resources are more readily available. 

### 5.2. Cytoreductive Surgery and HIPEC

For patients with limited peritoneal metastases in the absence of visceral or systemic metastases in colorectal cancer, cytoreductive surgery with hyperthermic peritoneal chemotherapy perfusion has been shown in a randomized study by Verwaal et al [35,36] to improve median survival from 12.6 months in patients receiving systemic chemotherapy alone to 22.4 months; these rates are comparable to those seen in more recent case control studies [36,37]. This may be under-estimating the efficacy of oxaliplatin-based chemotherapy. Subgroup analyses demonstrated vast differences in survival depending on volume of peritoneal disease [36]. In the context of limited operative resources, those with lower peritoneal cancer index (PCI) scores are more likely to obtain significant survival benefit from CRS/HIPEC, and should be considered over those with higher PCI scores. In order of most to least predicted benefit, patients may be stratified as having PCI < 10, PCI 10–20, and PCI > 20. Given poor outcomes in the more advanced group, PCI > 20 should be considered a contraindication for CRS/HIPEC [32,38]. 

Although the discussion regarding optimal regimen of HIPEC after CRS is ongoing [39], at present, the administration of HIPEC after cytoreductive surgery in the absence of limiting patient factors is standard management for patients with peritoneal metastases from CRC. Should CRS with HIPEC not be feasible in the context of individual OR and hospital resources, disposition for these patients and consideration of resumption of systemic chemotherapy as a bridge to surgery should be discussed in a multidisciplinary setting, whenever possible. While there are currently no studies examining such a treatment strategy specifically, one can extrapolate from randomized studies examining palliative chemotherapy treatment strategies. Specifically, those patients initially demonstrating chemosensitive tumours, who experience progression while on “chemo break,” can often be managed successfully with resumption of the same treatment [40]. For those patients in whom neoadjuvant chemotherapy was poorly tolerated, decisions regarding optimal timing of CRS/HIPEC versus alternative systemic options should be made in a multidisciplinary team on a case-by-case basis.

In the cases of unexpected incomplete cytoreduction, or completion of cytoreduction score greater than CC1 [26] (i.e., >2.5 mm size lesion or confluence remaining), the elimination of HIPEC should be strongly considered, weighing the potential morbidity against the minimal survival benefit in these patients when compared to systemic chemotherapy alone [41]. At present, given the absence of evidence supporting prophylactic HIPEC in patients without visible peritoneal metastatic disease, this should not be pursued during pandemic conditions [42]. 

## 6. Appendiceal Adenocarcinoma

The majority of the treatment for appendiceal adenocarcinomas is extrapolated from CRC data, given the rarity of this disease (0.12 per 1,000,000 people annually) [43]. As with M1c colorectal cancers, patients with intermediate and high-grade lesions—including exgoblet-cell carcinoids—are treated upfront with 3–6 months of neoadjuvant 5-fluorouracil-based chemotherapy in an attempt to diminish tumour burden, increase probability of complete cytoreduction, and ensure no development of visceral metastases in the interval preceding surgery [43,44,45,46]. In patients who are currently on systemic chemotherapy, we suggest continuation of this treatment as tolerated by the patient until surgical capacity is available.

The relationship between completeness of cytoreduction and outcome seen in colorectal cancers has been similarly demonstrated in retrospective studies examining appendiceal adenocarcinomas [18,46]. We suggest the principles for pursuing CRS with or without HIPEC as outlined for management of metastatic colorectal cancer (above) be applied to this pathologic group.

## 7. Peritoneal Mesothelioma

Peritoneal Mesothelioma (MPM) is a rare peritoneal malignancy arising from the peritoneum itself, with high propensity for morbidity and mortality due to significant local abdominal progression and predicted survival of less than 1 year without treatment [47,48]. Disease control rates ranging from stability to improvement in up to 71% of patients have been reported with systemic chemotherapy alone; however generally only result in a median overall survival of up to 10–26.8 months [48,49]. In comparison, a systematic review and meta-analysis performed by Helm et al (2015) reported CRS/HIPEC to confer a median overall survival in carefully selected patients of 19–92 months [48]. Despite the significant heterogeneity in histologic profiles and treatment protocols, these results support the pursuit of CRS/HIPEC when possible. Of note, 75% of patients received pre-operative systemic chemotherapy. Given the rarity of this disease, minimal data exists regarding optimal chemotherapy options for MPM; systemic treatment protocols are extrapolated from trials in pleural mesothelioma [50]. Adjuvant treatment protocols have, however, demonstrated superior progression free survival over surgery alone [51] and retrospective studies demonstrate short-term survival benefit from chemotherapy in both neoadjuvant and adjuvant settings [52]. Accordingly, for those patients likely to derive benefit from CRS/HIPEC in times of OR strain and limitation, neoadjuvant chemotherapy is a reasonable mitigating strategy until operative intervention can be performed. Importantly, these patients should be monitored closely for disease progression, so that more urgent prioritization can occur if concerns regarding resectability arise. 

Patient factors significantly associated with poorer prognosis and minimal benefit from CRS/HIPEC that may be used to stratify mesothelioma patients include: age >60 years, high-grade tumour biology, and incomplete cytoreduction (>CC1) [47]. In the context of limited resources, patients in whom CC2 cytoreduction (residual nodules 2.5 mm–2.5 cm after cytoreduction) is not possible should be preferentially treated non-operatively with systemic chemotherapeutic agents.

## 8. Special Considerations

### 8.1. Chemotherapy and COVID-19 Infection

In triaging and selecting treatment plans for patients with peritoneal disease, it is important to consider the risks associated with alternative treatment strategies utilized while surgical access is limited. Both chemotherapy and primary malignancy itself can cause systemic immunosuppression, and consequent increased susceptibility to viral infection, resulting in increased hospital admission and risk of mortality [53,54]. Specific to COVID-19 infection, data from a recent review of prospectively collected data in China suggested more frequent and more severe COVID-related complications in those who had undergone chemotherapy within the past month [12]. 

To mitigate risk of infection in these potentially immunocompromised patients, treatment protocols may be tailored by the medical oncology team to minimize exposure of patients to hospital settings where the likelihood of COVID-19 exposure is highest [55,56]. Practical considerations include telemedicine visits when appropriate, chemotherapy breaks, and substituting oral chemotherapy options where possible (i.e., Capecitabine for 5FU) which reduces time in the chemotherapy unit, negates the need for a central line, and minimises steroid doses [56].

### 8.2. Resource Utilization

When triaging patients in the context of a pandemic and strained health care resources, it is critical to consider the risk of the disease to the individual patient, the areas of capacity and deficiency within the individual health care setting, and how these often competing priorities can be balanced to offer the most benefit to the largest number of patients [1,3,4]. 

Studies examining post-operative morbidity and mortality from CRS/HIPEC report post-operative complications ranging from Clavien Dindo grades III–V in 24–49% of patients [29,39,57]. Complications include anastomotic leak, sepsis, fistula, abscess requiring intervention, thromboembolic events, and renal failure [58]; many of which may require admission to ICU. Mortality for patients undergoing CRS/HIPEC is 2–5% [25,26,44,59]. 

Early reports of CRS/HIPEC describe ICU stays of 7–20 days in the post-operative period [25]. While requirement for ICU, especially immediately following surgery has decreased as HIPEC has become more frequently utilized some retrospective studies note up to 67% of patients require ICU admission at some point during their hospital stay [20,59]. In cases of limited ICU resources, both upfront and delayed post-operative issues such as anastomotic leak potentially requiring higher level care must be considered and mitigated (e.g., lower threshold for diverting stoma in higher risk lower gastrointestinal anastomoses) when possible. Finally, hospitalization duration for CRS/HIPEC patients can be significant, with reported length of stays of 8–36 days (mean 6–13 days), including patients managed with enhanced recovery after surgery protocols [20,60].

A final resource potentially affected by the COVID-19 pandemic has been blood product availability. Due to closures of blood donation centers and social distancing practices, health systems are facing critical blood product shortages [61,62]. Large retrospective reviews demonstrate transfusion rates of up to 74% in patients undergoing CRS/HIPEC [63,64] suggesting blood product availability is a critical resource that must be considered prior to proceeding with these surgical cases in pandemic conditions.

## 9. Conclusions

The COVID-19 pandemic has strained hospital systems, and has resulted in triage and prioritization of surgical cases in an unprecedented way. For cancer patients specifically, this has required health care providers to weigh a number of intertwining factors that include not only disease process, but also the availability of temporizing alternative treatment strategies as well as system capacity to manage post-operative care. This review summarizes the available data regarding treatment strategies and the risk of progression of peritoneal malignancies to help inform and guide prioritization of these cases. While discussed in the context of the current pandemic, the strategies described in this review can also be applied to broader situations of restricted health care resources.

Triage of operative interventions for peritoneal disease is highly nuanced and depends significantly on individual site resources and challenges. Within the peritoneal disease population, consideration should be given to initial resource burden and predicted hospital stay, as well as the potential for additional critical care utilization should post-operative complications arise. Discussion in multidisciplinary contexts should be utilized frequently to aid in the prioritization and mitigating treatment strategies for individual patients as this dynamic situation continues to evolve. As capacity returns within systems, cases will require re-prioritization to ensure those requiring most urgent resection are given precedence; and hospitals will need to consider strategies to deal with the mounting backlog of delayed cancer cases. When resources are available, CRS and HIPEC should be pursued in line with pre-pandemic criteria, and remains the standard of care for the management of peritoneal disease. 

## Figures and Tables

**Table 1:** Summary of treatment approaches, mitigating strategies, and surveillance for peritoneal disease in pandemic conditions.

Pathology	Optimal Treatment	Mitigating Strategies	Prioritizing Features	Surveillance Required
Appendiceal Mucocele	Surgical Resection	Surgical delay	High risk features such as inflammation or regional lymphadenopathy	Cross sectional imaging to rule out progression or high risk features
PMP	CRS + HIPEC	Surgical delay Temporizing surgical debulking	Borderline resectability Symptoms from high disease burden or Krukenberg tumours	Q3 month cross sectional imaging to ensure no increased rate of progression suggestive of tumour dedifferentiation
CRC and Appendiceal Carcinoma Metastases	CRS + HIPEC + excision of primary for patients with PCI < 20 in whom CC0–CC1 CRS can be performed	Continue/return to neoadjuvant systemic chemotherapy	Threatened resectability Inability to tolerate chemotherapy Progression on chemotherapy	Q3 month cross sectional imaging and CEA levels to evaluate stability or progression of disease, and to monitor for visceral metastases
Mesothelioma	CRS + HIPEC for patients in whom CC0-CC2 CRS can be performed	Short term (<6 months) neoadjuvant systemic chemotherapy	Systemic treatment × 3–6 months completed Inability to tolerate chemotherapy Progression on chemotherapy	Q2 3 month cross sectional imaging for disease progression, including ascites

PMP: Pseudomyxoma Peritoneii; CRC: Colorectal Cancer; CRS: Cytoreductive Surgery; HIPEC: Hyperthermic Intraperitoneal Chemotherapy.; PCI: Peritoneal Cancer Index; CEA: Carcinoembryonic Antigen.

## References

[1] Spinelli A., Pellino G. (2020). COVID-19 pandemic: Perspectives on an unfolding crisis. Br. J. Surg..

[2] Brindle M., Gawande A. (2020). Managing COVID-19 in Surgical Systems. Ann. Surg..

[3] Rosenbaum L. (2020). Facing Covid-19 in Italy—Ethics, Logistics, and Therapeutics on the Epidemic’s Front Line. N. Engl. J. Med..

[4] Ueda M., Martins R., Hendrie P.C., McDonnell T., Crews J.R., Wong T.L., McCreery B., Jagels B., Crane A., Byrd D.R. (2020). Managing Cancer Care During the COVID-19 Pandemic: Agility and Collaboration Toward a Common Goal. J. Natl. Compr. Cancer Netw..

[5] Cancer Care Ontario (2020). COVID-19 Supplemental Clinical Guidance for Patients with Cancer. Dis. Manag. Resour..

[6] Society of Surgical Oncology Peritoneal Surface Malignancies Disease Site Work Group (2020). Resource for Management Options of Peritoneal Surface Malignancies During COVID-19. Dis. Site Specif. Manag. Resour..

[7] Finley C., Prashad A., Camuso N., Daly C., Aprikian A., Ball C.G., Bentley J., Charest D., Fata P., Helyer L. (2020). Canadian Society of Surgical Oncology. Guidance for management of cancer surgery during COVID-19 pandemic. Can. J. Surg..

[8] Truog R.D., Mitchell C., Daley G.Q. (2020). The Toughest Triage—Allocating Ventilators in a Pandemic. N. Engl. J. Med..

[9] Bartlett D.L., Howe J.R., Chang G., Crago A., Hogg M., Karakousis G., Levine E., Maker A., Mamounas E., McGuire K. (2020). Management of Cancer Surgery Cases During the COVID-19 Pandemic: Considerations. Ann. Surg. Oncol..

[10] Capobianco A., Cottone L., Monno A., Manfredi A.A., Rovere-Querini P. (2017). The peritoneum: Healing, immunity, and diseases. J. Pathol..

[11] Lei S., Jiang F., Su W., Chen C., Chen J., Mei W., Zhan L.Y., Jia Y., Zhang L., Liu D. (2020). Clinical characteristics and outcomes of patients undergoing surgeries during the incubation period of COVID-19 infection. EClinicalMedicine.

[12] Liang W., Guan W., Chen R., Wang W., Li J., Xu K., Li C., Ai Q., Lu W., Liang H. (2020). Cancer patients in SARS-CoV-2 infection: A nationwide analysis in China. Lancet Oncol..

[13] Angioni S. (2020). Laparoscopy in the coronavirus disease 2019 (COVID-19) era. Gynecol. Surg..

[14] National Comprehensive Cancer Network Coronavirus Disease 2019 (COVID-19) Resources for the Cancer Care Community. https://www.nccn.org/covid-19/.

[15] Tricco A.C., Antony J., Zarin W., Strifler L., Ghassemi M., Ivory J., Perrier L., Hutton B., Moher D., Straus S.E. (2015). A scoping review of rapid review methods. BMC Med..

[16] Kehagias I., Zygomalas A., Markopoulos G., Papandreou T., Kraniotis P. (2016). Diagnosis and Treatment of Mucinous Appendiceal Neoplasm Presented as Acute Appendicitis. Case Rep. Oncol. Med..

[17] Stocchi L., Wolff B.G., Larson D.R., Harrington J.R. (2003). Surgical treatment of appendiceal mucocele. Arch. Surg..

[18] Shaib W.L., Assi R., Shamseddine A., Alese O.B., Staley C., Memis B., Adsay V., Bekaii-Saab T., El-Rayes B.F. (2018). Appendiceal Mucinous Neoplasms: Diagnosis and Management. Oncologist.

[19] Sugarbaker P.H. (2006). The natural history, gross pathology, and histopathology of appendiceal epithelial neoplasms. Eur. J. Surg. Oncol..

[20] Cooksley T.J., Haji-Michael P. (2011). Post-operative critical care management of patients undergoing cytoreductive surgery and heated intraperitoneal chemotherapy (HIPEC). World J. Surg. Oncol..

[21] Foster J.M., Sleightholm R., Patel A., Shostrom V., Hall B., Neilsen B., Bartlett D., Smith L. (2019). Morbidity and Mortality Rates Following Cytoreductive Surgery Combined With Hyperthermic Intraperitoneal Chemotherapy Compared With Other High-Risk Surgical Oncology Procedures. JAMA Netw. Open.

[22] Zih F.S., Wong-Chong N., Hummel C., Petronis J., Panzarella T., Pollett A., McCart J.A., Swallow C.J. (2014). Mucinous tumor of the appendix with limited peritoneal spread: Is there a role for expectant observation?. Ann. Surg. Oncol..

[23] Chua T.C., Al-Zahrani A., Saxena A., Liauw W., Zhao J., Morris D.L. (2010). Secondary cytoreduction and perioperative intraperitoneal chemotherapy after initial debulking of pseudomyxoma peritonei: A study of timing and the impact of malignant dedifferentiation. J. Am. Coll. Surg..

[24] Miner T.J., Shia J., Jaques D.P., Klimstra D.S., Brennan M.F., Coit D.G. (2005). Long-term survival following treatment of pseudomyxoma peritonei: An analysis of surgical therapy. Ann. Surg..

[25] Chua T.C., Yan T.D., Smigielski M.E., Zhu K.J., Ng K.M., Zhao J., Morris D.L. (2009). Long-term survival in patients with pseudomyxoma peritonei treated with cytoreductive surgery and perioperative intraperitoneal chemotherapy: 10 years of experience from a single institution. Ann. Surg. Oncol..

[26] Sugarbaker P.H. (2009). Epithelial appendiceal neoplasms. Cancer J..

[27] Elias D., Gilly F., Quenet F., Bereder J.M., Sideris L., Mansvelt B., Lorimier G., Glehen O., de Chirurgie A.F. (2010). Pseudomyxoma peritonei: A French multicentric study of 301 patients treated with cytoreductive surgery and intraperitoneal chemotherapy. Eur. J. Surg. Oncol..

[28] Marcotte E., Sideris L., Drolet P., Mitchell A., Frenette S., Leblanc G., Leclerc Y.E., Dube P. (2008). Hyperthermic intraperitoneal chemotherapy with oxaliplatin for peritoneal carcinomatosis arising from appendix: Preliminary results of a survival analysis. Ann. Surg. Oncol..

[29] Chua T.C., Moran B.J., Sugarbaker P.H., Levine E.A., Glehen O., Gilly F.N., Baratti D., Deraco M., Elias D., Sardi A. (2012). Early- and long-term outcome data of patients with pseudomyxoma peritonei from appendiceal origin treated by a strategy of cytoreductive surgery and hyperthermic intraperitoneal chemotherapy. J. Clin. Oncol..

[30] Yan T.D., Bijelic L., Sugarbaker P.H. (2007). Critical analysis of treatment failure after complete cytoreductive surgery and perioperative intraperitoneal chemotherapy for peritoneal dissemination from appendiceal mucinous neoplasms. Ann. Surg. Oncol..

[31] Delhorme J.B., Elias D., Varatharajah S., Benhaim L., Dumont F., Honore C., Goere D. (2016). Can a Benefit be Expected from Surgical Debulking of Unresectable Pseudomyxoma Peritonei?. Ann. Surg. Oncol..

[32] Sanchez-Hidalgo J.M., Rodriguez-Ortiz L., Arjona-Sanchez A., Rufian-Pena S., Casado-Adam A., Cosano-Alvarez A., Briceno-Delgado J. (2019). Colorectal peritoneal metastases: Optimal management review. World J. Gastroenterol..

[33] Waite K., Youssef H. (2017). The Role of Neoadjuvant and Adjuvant Systemic Chemotherapy with Cytoreductive Surgery and Heated Intraperitoneal Chemotherapy for Colorectal Peritoneal Metastases: A Systematic Review. Ann. Surg. Oncol..

[34] Chicago Consensus Working Group (2020). The Chicago Consensus on peritoneal surface malignancies: Management of colorectal metastases. Cancer.

[35] Verwaal V.J., Bruin S., Boot H., van Slooten G., van Tinteren H. (2008). 8-year follow-up of randomized trial: Cytoreduction and hyperthermic intraperitoneal chemotherapy versus systemic chemotherapy in patients with peritoneal carcinomatosis of colorectal cancer. Ann. Surg. Oncol..

[36] Verwaal V.J., van Ruth S., de Bree E., van Sloothen G.W., van Tinteren H., Boot H., Zoetmulder F.A. (2003). Randomized trial of cytoreduction and hyperthermic intraperitoneal chemotherapy versus systemic chemotherapy and palliative surgery in patients with peritoneal carcinomatosis of colorectal cancer. J. Clin. Oncol..

[37] Elias D., Lefevre J.H., Chevalier J., Brouquet A., Marchal F., Classe J.M., Ferron G., Guilloit J.M., Meeus P., Goere D. (2009). Complete cytoreductive surgery plus intraperitoneal chemohyperthermia with oxaliplatin for peritoneal carcinomatosis of colorectal origin. J. Clin. Oncol..

[38] Faron M., Macovei R., Goere D., Honore C., Benhaim L., Elias D. (2016). Linear Relationship of Peritoneal Cancer Index and Survival in Patients with Peritoneal Metastases from Colorectal Cancer. Ann. Surg. Oncol..

[39] Quenet F., Elias D., Roca L., Goéré D., Ghouti L., Pocard M., Facy O., Arvieux C., Lorimier G., Pezet D. (2019). A UNICANCER phase III trial of Hyperthermic Intra-peritoneal Chemotherapy (HIPEC) for Colorectal Peritoneal Carcinomatosis. PRODIGE 7. Eur. J. Surg. Oncol..

[40] Maughan T.S., James R.D., Kerr D.J., Ledermann J.A., Seymour M.T., Topham C., McArdle C., Cain D., Stephens R.J., Medical Research Council Colorectal Cancer Group (2003). Comparison of intermittent and continuous palliative chemotherapy for advanced colorectal cancer: A multicentre randomised trial. Lancet.

[41] Glehen O., Kwiatkowski F., Sugarbaker P.H., Elias D., Levine E.A., De Simone M., Barone R., Yonemura Y., Cavaliere F., Quenet F. (2004). Cytoreductive surgery combined with perioperative intraperitoneal chemotherapy for the management of peritoneal carcinomatosis from colorectal cancer: A multi-institutional study. J. Clin. Oncol..

[42] Goere D., Glehen O., Quenet F., Ducreux M., Guilloit J.-M., Texier M., Benhamou E., Elias D. (2018). Results of a randomized phase 3 study evaluating the potential benefit of a second-look surgery plus HIPEC in patients at high risk of developing colorectal peritoneal metastases (PROPHYLOCHIP-NTC01226394). J. Clin. Oncol..

[43] Sideris L., Mitchell A., Drolet P., Leblanc G., Leclerc Y.E., Dube P. (2009). Surgical cytoreduction and intraperitoneal chemotherapy for peritoneal carcinomatosis arising from the appendix. Can. J. Surg..

[44] Levine E.A., Votanopoulos K.I., Shen P., Russell G., Fenstermaker J., Mansfield P., Bartlett D., Stewart J.H. (2018). A Multicenter Randomized Trial to Evaluate Hematologic Toxicities after Hyperthermic Intraperitoneal Chemotherapy with Oxaliplatin or Mitomycin in Patients with Appendiceal Tumors. J. Am. Coll. Surg..

[45] Lieu C.H., Lambert L.A., Wolff R.A., Eng C., Zhang N., Wen S., Rafeeq S., Taggart M., Fournier K., Royal R. (2012). Systemic chemotherapy and surgical cytoreduction for poorly differentiated and signet ring cell adenocarcinomas of the appendix. Ann. Oncol..

[46] Shapiro J.F., Chase J.L., Wolff R.A., Lambert L.A., Mansfield P.F., Overman M.J., Ohinata A., Liu J., Wang X., Eng C. (2010). Modern systemic chemotherapy in surgically unresectable neoplasms of appendiceal origin: A single-institution experience. Cancer.

[47] Alexander H.R., Li C.Y., Kennedy T.J. (2018). Current Management and Future Opportunities for Peritoneal Metastases: Peritoneal Mesothelioma. Ann. Surg. Oncol..

[48] Helm J.H., Miura J.T., Glenn J.A., Marcus R.K., Larrieux G., Jayakrishnan T.T., Donahue A.E., Gamblin T.C., Turaga K.K., Johnston F.M. (2015). Cytoreductive surgery and hyperthermic intraperitoneal chemotherapy for malignant peritoneal mesothelioma: A systematic review and meta-analysis. Ann. Surg. Oncol..

[49] Janne P.A., Wozniak A.J., Belani C.P., Keohan M.L., Ross H.J., Polikoff J.A., Mintzer D.M., Taylor L., Ashland J., Ye Z. (2005). Open-label study of pemetrexed alone or in combination with cisplatin for the treatment of patients with peritoneal mesothelioma: Outcomes of an expanded access program. Clin. Lung Cancer.

[50] Kindler H.L., Ismaila N., Armato S.G., Bueno R., Hesdorffer M., Jahan T., Jones C.M., Miettinen M., Pass H., Rimner A. (2018). Treatment of Malignant Pleural Mesothelioma: American Society of Clinical Oncology Clinical Practice Guideline. J. Clin. Oncol..

[51] Chicago Consensus Working Group (2020). The Chicago Consensus on peritoneal surface malignancies: Management of peritoneal mesothelioma. Cancer.

[52] Naffouje S.A., Tulla K.A., Salti G.I. (2018). The impact of chemotherapy and its timing on survival in malignant peritoneal mesothelioma treated with complete debulking. Med. Oncol..

[53] Kamboj M., Sepkowitz K.A. (2009). Nosocomial infections in patients with cancer. Lancet Oncol..

[54] Bitterman R., Eliakim-Raz N., Vinograd I., Zalmanovici Trestioreanu A., Leibovici L., Paul M. (2018). Influenza vaccines in immunosuppressed adults with cancer. Cochrane Database Syst. Rev..

[55] Burki T.K. (2020). Cancer guidelines during the COVID-19 pandemic. Lancet Oncol..

[56] You B., Ravaud A., Canivet A., Ganem G., Giraud P., Guimbaud R., Kaluzinski L., Krakowski I., Mayeur D., Grellety T. (2020). The official French guidelines to protect patients with cancer against SARS-CoV-2 infection. Lancet Oncol..

[57] Li X.B., Ma R., Ji Z.H., Lin Y.L., Zhang J., Yang Z.R., Chen L.F., Yan F.C., Li Y. (2020). Perioperative safety after cytoreductive surgery plus hyperthermic intraperitoneal chemotherapy for pseudomyxoma peritonei from appendiceal origin: Experience on 254 patients from a single center. Eur. J. Surg. Oncol..

[58] Rizvi S.A., Syed W., Shergill R. (2018). Approach to pseudomyxoma peritonei. World J. Gastrointest. Surg..

[59] Lopez-Basave H.N., Morales-Vasquez F., Mendez-Herrera C., Namendys-Silva S.A., Luna-Ortiz K., Calderillo-Ruiz G., Cabrera Rojas J., Ruiz-Garcia E., Herrera-Gomez A., Ruiz-Molina J.M. (2014). Intensive care unit admission after cytoreductive surgery and hyperthermic intraperitoneal chemotherapy. Is it necessary?. J. Oncol..

[60] Lu P.W., Fields A.C., Shabat G., Bleday R., Goldberg J.E., Irani J., Stopfkuchen-Evans M., Melnitchouk N. (2020). Cytoreductive Surgery and HIPEC in an Enhanced Recovery After Surgery Program: A Feasibility Study. J. Surg. Res..

[61] Shander A., Goobie S.M., Warner M.A., Aapro M., Bisbe E., Perez-Calatayud A.A., Callum J., Cushing M.M., Dyer W.B., Erhard J. (2020). The Essential Role of Patient Blood Management in a Pandemic: A Call for Action. Anesth. Analg..

[62] Pagano M.B., Hess J.R., Tsang H.C., Staley E., Gernsheimer T., Sen N., Clark C., Nester T., Bailey C., Alcorn K. (2020). Prepare to adapt: Blood supply and transfusion support during the first 2 weeks of the 2019 Novel Coronavirus (COVID-19) pandemic affecting Washington State. Transfusion.

[63] Saxena A., Valle S.J., Liauw W., Morris D.L. (2017). Allogenic Blood Transfusion Is an Independent Predictor of Poorer Peri-operative Outcomes and Reduced Long-Term Survival after Cytoreductive Surgery and Hyperthermic Intraperitoneal Chemotherapy: A Review of 936 Cases. J. Gastrointest. Surg..

[64] Nizri E., Kusamura S., Fallabrino G., Guaglio M., Baratti D., Deraco M. (2018). Dose-Dependent Effect of Red Blood Cells Transfusion on Perioperative and Long-Term Outcomes in Peritoneal Surface Malignancies Treated with Cytoreduction and HIPEC. Ann. Surg. Oncol..

